# Risk factors for bit‐related lesions in Finnish trotting horses

**DOI:** 10.1111/evj.13401

**Published:** 2021-01-28

**Authors:** Kati Tuomola, Nina Mäki‐Kihniä, Anna Valros, Anna Mykkänen, Minna Kujala‐Wirth

**Affiliations:** ^1^ Research Centre for Animal Welfare Department of Production Animal Medicine University of Helsinki Helsinki Finland; ^2^ Independent Researcher Pori Finland; ^3^ Department of Equine and Small Animal Medicine Faculty of Veterinary Medicine University of Helsinki Helsinki Finland; ^4^ Department of Production Animal Medicine Faculty of Veterinary Medicine University of Helsinki Helsinki Finland

**Keywords:** horse, animal welfare, bit, harness racing, oral lesion, trotter

## Abstract

**Background:**

Bit‐related lesions in competition horses have been documented, but little evidence exists concerning their potential risk factors.

**Objectives:**

To explore potential risk factors for oral lesions in Finnish trotters.

**Study design:**

Cross‐sectional study.

**Methods:**

The rostral part of the mouth of 261 horses (151 Standardbreds, 78 Finnhorses and 32 ponies) was examined after a harness race. Information on bit type, equipment and race performance was collected.

**Results:**

A multivariable logistic regression model of Standardbreds and Finnhorses showed a higher risk of moderate or severe oral lesion status associated with horses wearing a Crescendo bit (n = 38, OR 3.6, CI 1.4–8.9), a mullen mouth regulator bit (n = 25, OR 9.9, CI 2.2‐45) or a straight plastic bit (n = 14, OR 13.7, CI 1.75‐110) compared with horses wearing a snaffle trotting bit (n = 98, *P* = .002). Bar lesions (67 horses) were more common in horses wearing unjointed bits than in horses wearing jointed bits (Fisher's exact test *P* < .001). Lesions in the buccal area and the inner lip commissures were not associated with bit type. Using a tongue‐tie or an overcheck, galloping, placement in the top three or money earned in the race were not associated with lesion risk.

**Main limitations:**

The sample size for certain bit types was insufficient for statistical analysis.

**Conclusions:**

Moderate and severe oral lesion status was more common in horses wearing a Crescendo bit, a mullen mouth regulator bit or a straight plastic bit than in horses wearing a single‐jointed snaffle trotting bit. However, lesions were observed regardless of bit type. Further studies on rein tension, the interaction between bit type and rein tension and prevention of mouth lesions in trotters are warranted.

## 1 INTRODUCTION

Bit‐related lesions, causing pain and diminishing equine welfare, are common in competition horses.^1^, ^2^, ^3^, ^4^, ^5^, ^6^, ^7^ In Nordic countries, an 84%‐88% occurrence of oral lesions in the bit area after racing has been reported.^1^, ^5^ Only few studies have described bit types as a risk factor; in Icelandic horses, a curb bit with a port was associated with a higher risk of lesions in the bars of the mandible compared with a snaffle bit or a traditional Icelandic curb bit,^3^ and a snaffle bit was associated with a higher risk for buccal lesions compared with the various curb bits.^3^ Snaffle‐bitted racehorses had more lesions than did gag‐bitted polo ponies,^2^ and 11‐mm Myler‐bitted ridden horses were less stressed and expressed less head‐tossing than horses ridden with a traditional 18‐mm snaffle bit.^8^ Unridden horses on a treadmill independently applied higher rein tension with a double‐jointed bit compared with an unjointed but curved mullen mouth snaffle bit.^9^

Horses can be controlled without a bit during exercise,^10^ but bitting is considered an obligatory safety measure in harness racing. Successful bit use is based on the principles of negative reinforcement, and problematic behavioural consequences of erroneously applied pressure or pain are common and well described elsewhere (see eg 11, 12). Scientific literature is still scarce on identifying the risks posed by bits and tack in a competition setting, yet developing means to minimise work‐related lesions is necessary for welfare and ethical reasons.^13^, ^14^, ^15^ The aim of this study was to investigate whether mouth lesions in a mixed population of Finnish trotting horses were associated with certain bits, trotter's equipment or race performance.

## 2 MATERIALS AND METHODS

### 2.1 Horses and oral examination

The data were collected from horses as previously reported,^1^ as part of a welfare programme for trotters conducted by The Finnish Trotting and Breeding Association (Suomen Hippos ry) in 2017. A total of 261 horses were evaluated. These were privately owned trotters participating in 10 separate harness racing events on four racetracks in western Finland. As described previously,^1^ the horses were initially selected at random from the starting lists. Priority was occasionally given to first arrivers at the harnessing booths to ensure collection of an adequate sample size in the limited timeframe between races.

The rostral part of the oral cavity was examined systematically.^1^ Lesion location was identified as inner lip commissures, outer lip commissures, bars of the mandible, buccal area near the second premolar tooth (106, 206), tongue or hard palate.^1^ Points were given for each acute lesion. Bruises (submucosal bleeding) were given points according to their size as follows: <0.5 cm = 1 point; 0.5−1 cm = 2 points; >1 cm but <3 cm = 3 points; ≥3 cm = 4 points. Wounds (mucosal surface damaged) were given points as follows: <0.5 cm = 2 points; 0.5−1 cm = 4 points; >1 cm but <3 cm = 6 points; ≥3 cm = 8 points. For deep wounds, additional two points were added.^1^ For each horse, points were summed up to obtain a total lesion score, which determined the severity category as follows: A (no acute lesions), horses with 0 points; B (mild lesion status), horses with 1‐2 points; C (moderate lesion status), horses with 3‐11 points, but excluding horses with eight points from one single lesion; and D (severe lesion status), horses with 12 or more points and horses with eight points from one single lesion.^1^ For statistical analysis, lesion severity categories A‐D were merged into two categories: AB (no lesions or mild lesion status) and CD (moderate or severe lesion status). From a clinical point of view, the most severe case in the combined AB group was a horse with two points (eg a horse with one bruise not exceeding 1 cm). Horses with more severe lesions fell into the combined CD group, where the lower cut‐off limit was three points (eg a horse with one bruise exceeding 1 cm) and in this data set, the maximum case was 36 points (a horse with two wounds exceeding 1 cm, one wound equal or exceeding 3 cm, one deep wound exceeding 1 cm and two wounds not exceeding 1 cm in different locations). Additionally, the presence or absence of blood inside the mouth was recorded.^1^ Old lesions (scars, depigmentation of outer lip commissures, old bruises and old wounds) were recorded separately and excluded from the analysis.

### 2.2 Data collection

The breed, age and sex of the horse were recorded along with bit type, mouthpiece material and bit thickness, which was measured adjacent to the bit ring with a vernier caliper. Additional variables recorded were overcheck (yes/no), check bit (yes/no), check bit type, jaw strap (yes/no), tongue‐tie (yes/no) and tongue‐tie material. The following variables were obtained from Heppa database (Suomen Hippos ry's online database for information on horses and racing): start type (auto start or volt start), race distance (1100 m (Shetland ponies only), 1600 m, 2100 m or 2600 m), whether the horse won money in the race (yes/no), was placed in the top three (yes/no) or galloped during the race (yes/no) and whether the horse had raced within the last 2 weeks (yes/no). Additionally, the driver, the trainer and their license types were recorded.

### 2.3 Data analysis

Data were analysed statistically using Stata IC version 16 (Stata Corporation). Univariable analyses of the associations between all potential risk factors and outcome variable of interest (AB vs CD lesion status) were first computed using Chi‐square tests (Table S1). Three age groups were formed (3‐5, 6‐9 and 10‐15 years). For bit thickness, four categories were created: thin (12‐13 mm), basic (14‐17 mm), thick (18‐22 mm) and extra thick (23‐30 mm) bits.

Relationships between potential risk factors were evaluated by examining pair‐wise associations and were taken into consideration when building the logistic regression model. Associations were considered significant if *P* < .05. Bit type, but not bit thickness, was included in the model as there was an association between them, with all unjointed bits being thick or extra thick, and bit type was our main variable of interest. Breed was associated with bit type. Compared with Standardbreds, Finnhorses were more often bitted with a Crescendo or a mullen mouth regulator bit. Parallel models were run with and without breed to ensure that the association between breed and bit type did not affect the results. The outcome of these models was very similar for all the other risk factors. Thus, as breed was considered biologically important, the model including breed is reported, despite this association. The driver license type was associated with the breed: most Standardbreds were driven by drivers with Licence A, and Finnhorses by drivers with Licence B or C.

Pony drivers are typically a minor with a pony licence. The reported pony results are descriptive only and excluded from statistical analysis due to small sample size, over‐representation of the snaffle trotting bit (21 of 32) and the fundamental difference between betting and nonbetting races, in which ponies participate exclusively.

**FIGURE 1:**
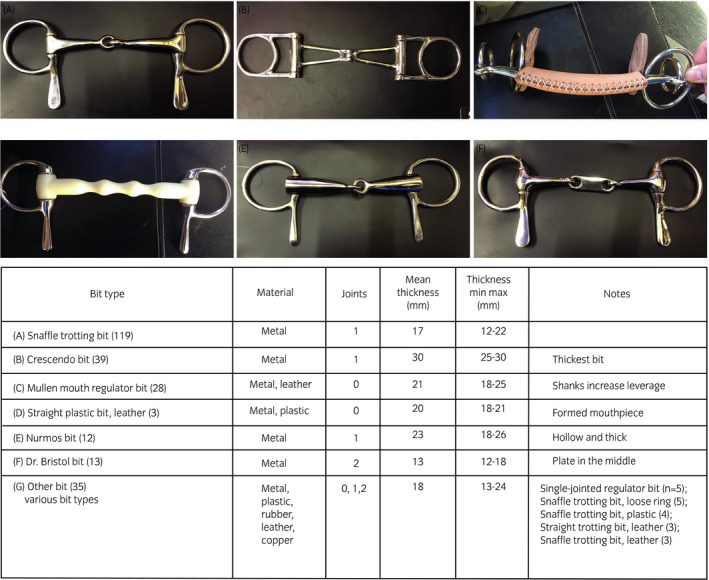
Six most common bits used on trotters in the study

The six most common bit types and the group ‘other bit’ were included in the model (Figure 1). Nurmos bits (n = 10) and moisturiser bits (n = 2) were combined due to their similarity and collectively named the Nurmos bit group. All horses with a straight plastic bit (vernacular: happy mouth bit or apple bit, n = 14) had a CD lesion status. One of these horses received three points (one < 0.5 cm wound and one < 0.5 cm bruise at the bars) and was thus very close to the B lesion status cut‐off limit. This horse was moved into the AB group to enable the logistic regression analysis. Manual stepwise backward and forward procedures were used to build the model, and explanatory variables, except breed, were eliminated until all remaining parameters had an association with a *P*‐value of ≤.05. At each step, the removed variables were evaluated for confounding effects by checking whether the coefficients for the remaining variables changed substantially. All relevant interactions (eg sex × age, distance × breed, distance × age, bit type × bit thickness, bit type × tongue‐tie and bit type × overcheck) were tested one by one, but no significant association with CD lesion status was detected.

The number of horses included in the model was 229. Horses were trained by 171 individual trainers and driven by 120 individual drivers. The majority of them trained or drove only one horse. The model was tested with the trainer or driver as a random factor and both proved nonsignificant. This was expected considering a large number of trainers and drivers. The final model, containing breed, sex and bit type, is presented as a simple multivariable logistic regression model. The model was evaluated by a sensitivity and specificity test, ROC curve inspection, the goodness of fit test and by evaluating the residuals per covariate pattern and influential data (leverage and delta‐betas). Results of the final model, in Table 1, are maximum likelihood estimates and presented as odd ratios (OR) and 95% confidence interval (CI). The association between lesion location and blood detected with any given bit type was analysed with the Fisher's exact test. The association between blood in the mouth and breed was analysed with the Pearson Chi‐square test. Significance was set at *P* ≤ .05.

**TABLE 1:** The results of multivariable logistic regression model. Risk factors for moderate or severe oral lesion status (CD) compared with no lesions or mild lesion status (AB). N = 229

Variable	Category	n	Horses in CD group (%)	OR	95% CI	*P*‐value
Breed						.3
	Standardbred	151	92 (61)	Reference		
	Finnhorse	78	58 (74)	1.5	0.8‐2.9	
Sex						.05
	Gelding	98	56 (57)	Reference		
	Mare	102	75 (74)	2.2	1.2‐4.2	.01
	Stallion	29	19 (66)	1.3	0.5‐3.4	.5
Bit type						.002
	Snaffle trotting	98	49 (50)	Reference		
	Crescendo	38	30 (79)	3.6	1.4‐8.9	.007
	Mullen mouth regulator	25	23 (92)	9.9	2.2‐45.2	.003
	Straight plastic	14	13 (93)	13.7	1.7‐110	.01
	Nurmos	12	6 (50)	1.1	0.3‐3.6	>.9
	Dr. Bristol	10	8 (80)	3.9	0.8‐20	.1
	Other	32	20 (63)	1.7	0.7‐3.9	.2

## 3 RESULTS

### 3.1 Logistic regression model

Of the 229 horses, 151 were Standardbreds and 78 were Finnhorses; 102 were mares, 98 were geldings and 29 were stallions and age ranged from 3 to 15 years (Mean 6.9, SD 2.6). Of the Standardbreds, 83% (125/151) had acute lesions; and their lesion status distribution was 17% (26/151) A status, 22% (33/151) B status, 43% (65/151) C status and 18% (27/151) D status. Of the Finnhorses, 90% (70/78) had acute lesions with a distribution of 10% (8/78) A status, 17% (13/78) B status, 44% (34/78) C status and 29% (23/78) D status.

The full model containing breed, sex and bit type was statistically significant (N = 229, *χ* ^2^ 38.75, *P* < .001) indicating that the model was able to distinguish between AB and CD lesion status horses. The model's sensitivity was 83% and specificity was 42%. The model correctly classified 68% of the cases. The area under the ROC curve was 73% (CI 67%‐80%, *P* < .001). The *P*‐value for the Pearson *χ* ^2^ goodness of fit test was 0.4.

**FIGURE 2:**
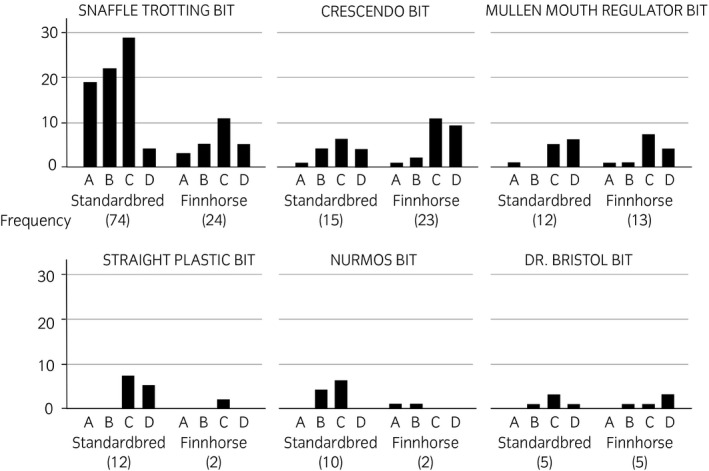
Standardbreds and Finnhorses according to bit type and lesion category. No lesions (A), mild (B), moderate (C) and severe (D) lesion status (N = 229)

**FIGURE 3:**
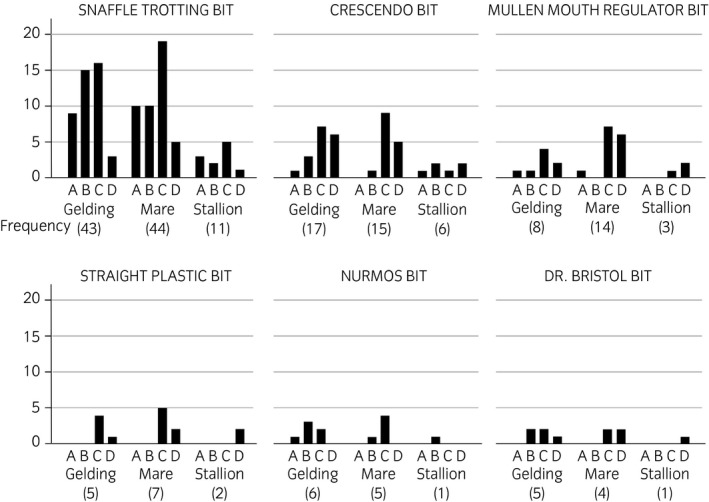
Geldings, mares and stallions according to bit type and lesion category. No lesions (A), mild (B), moderate (C) and severe (D) lesion status (N = 229)

The snaffle trotting bit was the most common bit among all the breeds (Figure 2). The CD lesion status was recorded for 50% (49/98) of horses wearing the snaffle, but the risk of CD lesion status was higher for horses wearing a Crescendo bit 79% (30/38), a mullen mouth regulator bit 92% (23/25) or a straight plastic bit 100% (14/14) (*P* = .002) (Table 1). Bit thickness was not associated with CD lesion status. Sex was associated with CD lesion status (*P* = .05). Mares had a higher risk for CD lesion status than did geldings but stallions (only 29 horses) did not differentiate from geldings (Table 1, Figure 3). Breed, retained in the model, was not significant.

### 3.2 Lesion location, blood and bit type

Unjointed bits were associated with the occurrence of bar lesions (67 horses). Bar lesions were found in 86% (12/14) of the horses wearing a straight plastic bit, in 64% (16/25) of the horses wearing a mullen mouth regulator bit and in 50% (6/12) of the horses wearing a Nurmos, but only in 20% (2/10) of the horses wearing a Dr. Bristol bit, in 19% (19/98) of the horses wearing a snaffle trotting bit and in 8% (3/38) of the horses wearing a Crescendo bit (*P* < .001). Bit type was associated neither with lesions in the buccal area (63 horses) (*P* > .9) nor the lesions in the inner lip commissures (145 horses) (*P* = .2). For the lesion location analysis, we only considered whether the horse had lesions in a particular location while disregarding the severity and number of lesions. The low number of lesions in the outer lip commissures (16 horses), the tongue (nine horses) and the hard palate (one horse) did not allow for analysis of their association with bit type. Blood in the mouth was present more often in Finnhorses (21%, 16/78) than in Standardbreds (10%, 15/151, *P* = .03). Number of blood observations per bit type was as follows: Crescendo bit 10/38, straight a plastic bit 3/14, Dr. Bristol bit 2/10, mullen mouth regulator bit 4/25, other bit 4/32, snaffle trotting bit 8/98 and Nurmos bit 0/12.

### 3.3 Equipment and race performance

Using a tongue‐tie or overcheck was not associated with CD lesion status in univariable analysis, so they were not included in the logistic regression model (Table S1). A tongue‐tie was fitted on 72% of the horses (166/229), with most common materials being an elastic leg bandage (111 horses), Vet Flex or Vet Wrap (28 horses) or stockings (15 horses). An overcheck was recorded for 83% of Standardbreds (125/151) and 96% of Finnhorses (75/78). Among these, a jaw strap was used on 44% of horses, a check bit on 44% of horses and both of them on 12% of horses. The most common check bit type was a straight basic check bit.

Galloping during the race, placement among the top three, money earned in the race, race distance, start type or racing previously no longer than 2 weeks ago were not lesion risk factors. License types, eight for trainers and three for drivers, were not associated with the occurrence of CD lesion status (Table S1).

### 3.4 Trotting ponies

In total 32 ponies were examined, 18 Shetland ponies and 14 Gotland Russ ponies. Lesion status A was described for 25% (8/32), B status for 28% (9/32), C status for 44% (14/32) and D status for 3% (1/32) of the ponies. Eight ponies (25%) had wounds and 23 (72%) had bruises. Blood was observed in the mouth of one pony. Twenty‐one ponies wore a snaffle trotting bit, and less common bit types were the Crescendo (n = 1), straight plastic (n = 1), mullen mouth regulator (n = 1), Dr. Bristol (n = 3) and other bit (n = 3). Of all ponies, 9% (3/32) had a tongue‐tie and 88% (28/32) raced with an overcheck.

## 4 DISCUSSION

Bit type was a risk factor for CD oral lesion status, yet our model does not allow for determining any fractions of this total risk to a particular cause, such as rein tension, action mechanics or physical properties of the bit (such as material‐specific friction or bit form) or their interaction. The Crescendo bit is commonly considered the most ‘severe’ bit of our sample with its thin metal rails presumably focusing pressure on a relatively small contact area in the mouth. Horses racing with this bit had an elevated risk of CD lesion status compared with horses racing with snaffle trotting bit. Compared with the Crescendo, the mullen mouth regulator and unjointed bits are often considered ‘gentler’, but horses bitted with these also carried an elevated risk of a CD lesion status. The leather covering and thickness of the mullen mouth regulator bit may contribute to a ‘gentler’ appearance, but the shanks may in fact amplify rein tension through their lever action.^16^ The jointed snaffle bit is generally regarded as a ‘gentle bit’.^17^ However, CD lesion status occurred in half of the horses racing with the single‐jointed snaffle trotting bit and the Nurmos bit. A minor finding in the current study—potentially affecting the mechanical action of the bit—was that, six bits were fitted on backwards accidentally (two trainers) and on purpose (four trainers) on the rationale that the bit would have a less severe effect. Harness racing guidelines in Finland state that all equipment should be correctly fitted but fail to describe the correct fit.^18^

**FIGURE 4:**
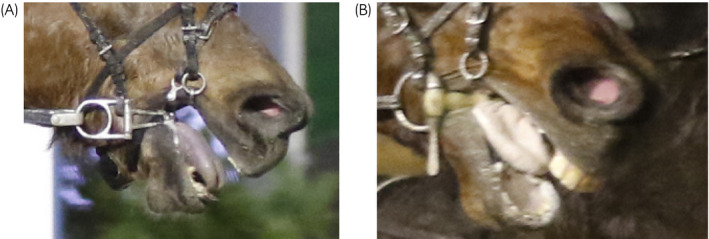
A, A horse racing with Crescendo bit. Rein tension is applied to the bit and the bit is compressing lip commissures and tongue. B, A horse racing with straight plastic bit. Rein tension is applied to the bit and the bit is compressing lip commissures and bars of the mandible (Copyrighted image).

In the current study, horses wearing unjointed bits had more bar lesions than horses wearing jointed bits. The bars, a thinly covered bony structure under the bit, are particularly vulnerable to trauma.^19^ It seems that the potent action of an unjointed bit, in particular, may compress the mucous membrane causing ulceration adjacent to the first lower cheek teeth (Figure 4). Large and painful lesions adjacent to the first lower cheek teeth and even traumatised mandibular bone receding from the reserve crown have been found in a previous study of trotting horses.^20^ Another study has documented bar lesions in half (51%) of competing Icelandic horses wearing curb bits with ports but the ratio between unjointed and jointed bits was not reported.^3^

Bit choice is mainly based on the subjective assessment by the trainer or driver.^19^ It has been suggested that ‘the multiplicity of bits now on the market strongly suggest that bit designs are used to overcome training and performance issues, many of which probably reflect some deficits in training or riding’.^16^ Horses that do not respond to light rein signals are a common issue in equitation and are called ‘pullers’, ‘heavy‐mouthed’ or ‘hard‐mouthed’.^12^, ^20^ In training, negative reinforcement is usually used to teach the horse to respond in a certain manner to rein signals, also called aids or cues, which usually involve applying pressure to the bit via rein tension.^21^ It is possible that Crescendo, mullen mouth regulator and unjointed happy mouth bits are chosen for horses that are unresponsive to light rein signals for various reasons, including an evasion or flight response due to painful stimulus or anticipation of pain,^22^, ^23^ habituation to bit pressure due to inconsistent training not reinforcing the desired reaction to bit pressure^12^, ^16^ and multiple stressors present in the competition environment such as transportation, unfamiliar horses and novel situations.^24^, ^25^ Inability to respond to a light rein signal due to erroneous learning combined with high arousal (be that excitement, anxiety or fear) may increase the rein tension needed and predispose these horses to oral trauma. As there might be complicated interactions between factors such as horse behaviour and performance, and bit type and rein tension, it would be interesting to follow the same horses driven with different bits and different rein tensions.

Sex was unexpectedly associated with lesion risk, so that mares compared with geldings were at higher risk of CD lesion status. Horse handlers need to note this risk potential. This finding supports further studies on sex differences, as existing literature recognises sex‐based attitudes potentially affecting horse handling, such as mares assumed more anxious and flighty than geldings and anthropomorphically gender‐stereotyping mares as ‘difficult’.^26^, ^27^

Other variables did not prove significant risk factors for lesions. Of the horses, 26% galloped during the race. Once a horse gallops, the driver usually pulls on the reins to slow the horse down to a trot, but in contrast to our expectations, no association emerged between galloping and lesion severity.

Tongue‐ties were used on the majority of horses. Use of a tongue‐tie is allowed in racing in Finland, in contrast to Switzerland and parts of Germany, where its use is forbidden.^28^ Finnish racing rules do not regulate tongue‐tie width in contrast with Sweden, where the tongue‐tie must be at least 10‐mm wide.^18^, ^29^ In the current study, the tongue‐tie was not associated with lesions.

Good performance does not guarantee good welfare, even though that still is a common belief.^30^ Horses with a CD lesion status were placed in the top three or earned money in the race similarly to horses with a AB lesion status. Lesions, although potentially painful, do not necessarily manifest in concurrent poor performance, because pain sensation might be temporarily suppressed by stress‐induced analgesia under stressful conditions.^31^ Currently, it is not fully understood how negative experiences from lesions are linked to the horse's behaviour later on during their competition career nor is the safety risk for humans fully appreciated.^22^, ^32^ However, learning and mood are affected by all experiences^11^, ^12^ and pain or discomfort can elicit a fear reaction, acute stress response and, later, anticipatory stress in the competition environment.^11^, ^25^, ^32^, ^33^ ‘Flightiness’ is a trait that some might consider advantageous to a racehorse to a certain degree,^11^ but it can constitute risk for accidents.

The current study has some limitations. Firstly, the number of certain bit types was too small for robust statistical results. The group using straight plastic bits comprised only 14 horses, so confirming the result requires further research in a larger horse population. Secondly, post‐racing examination does not determine the exact moment of lesion occurrence. Only acute lesions were included in this analysis, but obviously some lesions might have been present before the race, for example, due to different bit used in training. However, if this was the case, it would only further emphasise the need for racing awareness on and control of the oral health of trotters. Thirdly, horses were selected at random but not randomly in a statistical sense, in order to maximise the number of horses examined in the limited timeframe between races. Among those not examined previously, priority was occasionally put on those horses first to arrive to their harnessing booth. First arrivers might be horses that did not finish or were disqualified, but only 34 horses (15%) were such cases in the current study. Finally, in a previous Danish study, noseband use has been associated with lip commissure lesions.^7^ Noseband tightness was not measured in the current study due to limited data collection timeframe.

Only one study has evaluated rein tensions in trotters previously. Maximum rein tensions among trotting horses were twice as high as in riding horses.^34^ Several studies on rein tension are available for ridden horses.^17^, ^35^, ^36^, ^37^ Horses are not voluntarily willing to tolerate great or prolonged rein tension in exchange for rewards, and rein tension has been correlated with expression of conflict behaviour, such as mouth gaping (Figure 4),^24^, ^35^, ^36^, ^38^ leading to reduced rideability or analogously reduced driveability.^36^ It would be useful to assess the association of oral lesions, conflict behaviour and rein tension in trotters as well.

In conclusion, crescendo, mullen mouth regulator or straight plastic bitted trotters had a higher risk of moderate or severe oral lesion status after a race than horses racing with single‐jointed snaffle trotting bits, but lesions occurred regardless of bit type. Horses racing with unjointed bits had more bar lesions than horses racing with jointed bits. Further studies on rein tension, the interaction between bit type and rein tension and prevention of mouth lesions in trotters are warranted.

## CONFLICT OF INTERESTS

The authors declare that this study received funding from Suomen Hippos ry. The data were collected during a welfare programme for trotters, conducted by Suomen Hippos ry. The funder approved the proposed data collection method but had no further role in the study design, collection, analysis or interpretation of the data or preparation of the manuscript. The decision to submit the report for publication was made by the authors and was approved by the funder. K. Tuomola works as a race veterinarian at Porin Ravit Oy, which is one of the tracks where horses were examined, but she was not on duty during the research period. All authors declare no conflict of interest.

## AUTHOR CONTRIBUTIONS

K. Tuomola contributed to the study design, data collection and analyses and preparation of the manuscript. M. Kujala‐Wirth performed the statistical analysis. N. Mäki‐Kihniä contributed to data collection and analyses, and manuscript preparation. M. Kujala‐Wirth, A. Valros and A. Mykkänen contributed to interpreting the results and manuscript preparation. K. Tuomola and M. Kujala‐Wirth have had a full access to all data in the study and take responsibility for the integrity of the data and the accuracy of the data analysis. All authors have read and approved the final manuscript.

## ETHICAL ANIMAL RESEARCH

The study was considered ethically acceptable by the University of Helsinki Viikki Campus Research Ethics Committee (Statement 8/2018).

## INFORMED CONSENT

The oral examination was compulsory for participants in the harness racing events. Suomen Hippos ry informed the trainers of the study on their website (www.hippos.fi) and in their newspaper (Hevosurheilu) prior to the study.

### Peer Review

The peer review history for this article is available at https://publons.com/publon/10.1111/evj.13401.

## Supporting information

Table S1

Infographic

Poruguese Summary

## Data Availability

The data that support the findings of this study are available from the corresponding author upon reasonable request.
