# Supplementary material for: Risk factors for bit‐related lesions in Finnish trotting horses
Source: Equine Vet J. 2021 Jan 28;53(6):1132–40. doi: 10.1111/evj.13401 (PMC8518388; doi:10.1111/evj.13401)
Supplement: Supplementary file 1 — Table S1 [file EVJ-53-1132-s003.pdf]

Equine Veterinary Journal BEVA

Table S1: The results of the univariable analysis (Chi-square test and Fisher's exact test in bit type) in Standardbreds and Finnhorses. Summary of trotter's explanatory variables and their association with an outcome; moderate or severe oral lesion status (CD) vs. no lesions or mild oral lesion status (AB). From all Standardbreds and Finnhorses 66% (150/229) had CD lesion status. P-value < 0.05 was considered significant (N = 229).

Variable	Category	n	Horses in CD group	(%)	P-value
**Breed**					0.04
	Finnhorse	78	58	(74)	
	Standardbred	151	92	(61)	
**Sex**					0.05
	Mare	102	75	(74)	
	Stallion	29	19	(66)	
	Gelding	98	56	(57)	
**Age (years)**					0.4
	3–6	119	82	(69)	
	7–9	74	44	(59)	
	10–15	36	24	(67)	
**Overcheck**					0.2
	Yes	200	134	(67)	
	No	29	16	(55)	
**Check bit**					0.7
	Yes	110	71	(65)	
	No	117	78	(67)	
**Jaw strap**					0.3
	Yes	112	77	(69)	
	No	116	72	(62)	
**Check bit and jaw strap**					> 0.9
	Yes	23	15	(65)	
	No	204	134	(66)	
**Tongue-tie**					0.8
	Yes	166	110	(66)	
	No	62	40	(65)	
**Bit type**					< 0.001
	Snaffle trotting	98	49	(50)	
	Crescendo	38	30	(79)	
	Mullen mouth regulator	25	23	(92)	
	Straight plastic	14	14	(100)	
	Nurmos	12	6	(50)	
	Dr. Bristol	10	8	(80)	
	Other	32	20	(63)	
**Bit thickness (mm)**					0.5
	10–13	14	9	(64)	
	14–17	60	35	(58)	
	18–22	104	69	(66)	
	23–30	51	37	(73)	
**Galloping during the race**					0.7
	Yes	59	40	(68)	
	No	170	110	(65)	
**Placement in the top three**					0.2
	Yes	60	35	(58)	
	No	169	115	(68)	
**Money earned in the race**					0.3
	Yes	124	85	(69)	
	No	105	65	(62)	
**Start type**					0.3
	Auto	150	95	(63)	
	Volt	79	55	(70)	
**Race distance (m)**					0.2
	1600	63	44	(70)	
	2100	156	102	(65)	
	2600	10	4	(40)	
**Competed in last 14 days**					0.8
	Yes	107	69	(64)	
	No	122	81	(66)	
**Driver's license type**					0.2
	A (the most experienced)	185	116	(63)	
	B	22	16	(73)	
	C	22	18	(82)	
**Trainer's license type**					0.7
	Professional license	92	59	(64)	
	Other license	137	91	(66)	
