# Supplementary material for: Risk factors for bit‐related lesions in Finnish trotting horses
Source: Equine Vet J. 2021 Jan 28;53(6):1132–40. doi: 10.1111/evj.13401 (PMC8518388; doi:10.1111/evj.13401)
Supplement: Supplementary file 2 — Infographic [file EVJ-53-1132-s001.pdf]

Risk factors for bit-related lesions in Finnish trotting horses

Equine Veterinary Journal
BEVA Journals

Kati Tuomola, Nina Mäki-Kihniä, Anna Valros, Anna Mykkänen, Minna Kujala-Wirth

@ kati.tuomola@helsinki.fi Research Centre for Animal Welfare, Department of Production Animal Medicine, University of Helsinki, Finland

Risk factors for bit-related lesions in Finnish trotting horses

First published 17 December 2020
doi:10.1111/evj.13401
https://beva.onlinelibrary.wiley.com/doi/abs/10.1111/evj.13401

RISK FACTORS FOR MODERATE OR SEVERE MOUTH LESION STATUS (CD) with odds ratio OR

BIT TYPE

SNAFFLE TROTTING BIT (REFERENCE)

Odds ratio ⊢ 95%CI ⊣

n = 98, OR 1.0

CD 50%

Occurrence of CD lesion status in horses racing with this bit type

CRESCENDO

n = 38, OR 3.6 (1.4–8.9)

CD 79%

MULLEN MOUTH REGULATOR

n = 25, OR 9.9 (2.2–45.2)

CD 92%

STRAIGHT PLASTIC

n = 14, OR 13.7 (1.7–110)

CD 93%

SEX

REFERENCE

GELDING: n = 98, OR 1.0, CD 57%

MARE: n = 102, OR 2.2 (1.2–4.2), CD 74%

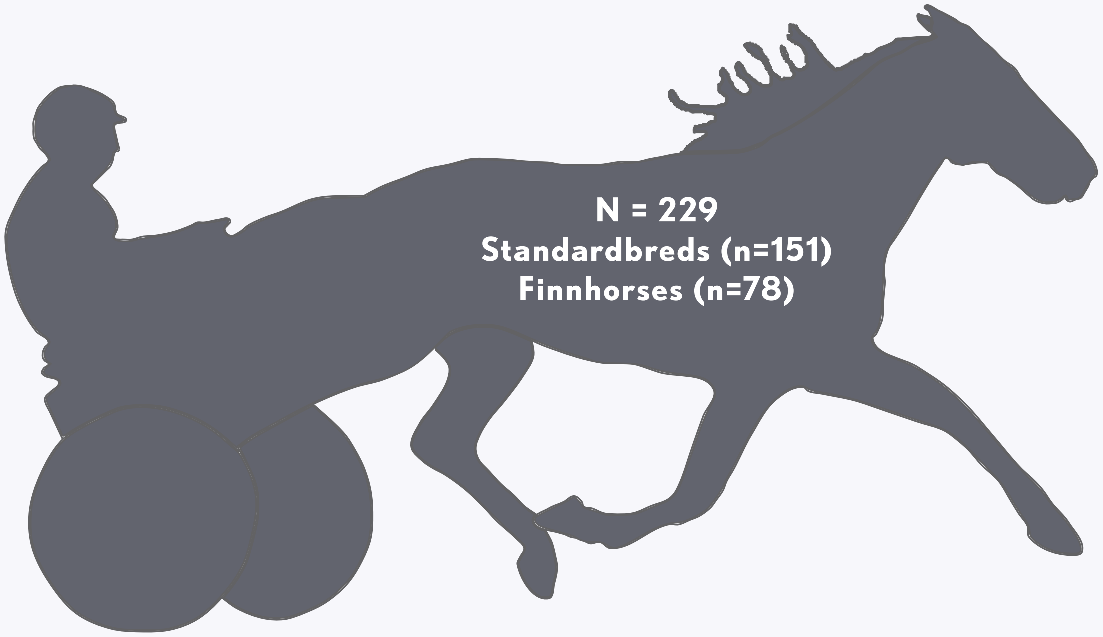

RISK FACTOR FOR BAR LESIONS

UNJOINTED BIT-TYPES vs. JOINTED BIT-TYPES (REFERENCE)

Unjointed bit-types: Risk factor for bar lesions, $P < .001$

NO ASSOCIATION FOUND

WITH LESION STATUS

- TONGUE TIE
- OVERCHECK
- GALLOPING
- RACE PERFORMANCE
- BREED
