# Supplementary material for: Risk factors for bit‐related lesions in Finnish trotting horses
Source: Equine Vet J. 2021 Jan 28;53(6):1132–40. doi: 10.1111/evj.13401 (PMC8518388; doi:10.1111/evj.13401)
Supplement: Supplementary file 3 — Poruguese Summary [file EVJ-53-1132-s002.docx]

**Fatores de risco de lesões associadas à embocadura em cavalos Finlandeses de trote**

Kati Tuomola*^1^, Nina Mäki-Kihniä^2^, Anna Valros^1^, Anna Mykkänen^3^ and Minna Kujala-Wirth^4^

^1^Research Centre for Animal Welfare, Department of Production Animal Medicine, University of Helsinki, Finland;

^2^Independent researcher, Pori, Finland;

^3^Department of Equine and Small Animal Medicine, Faculty of Veterinary Medicine, University of Helsinki, Finland and

^4^Department of Production Animal Medicine, Faculty of Veterinary Medicine, University of Helsinki, Finland.

*** email do autor para correspondência:** [kati.tuomola@helsinki.fi](mailto:kati.tuomola@helsinki.fi)

**Palavras-chave:** Bem-estar animal, embocadura, corrida de arreios, cavalo, lesão oral, trotador.

**Título curto:** Fatores de risco de lesões associadas à embocadura em trotadores Finlandeses

**Resumo**

**Introdução:** Lesões associadas ao uso de embocaduras em cavalos de competição têm sido documentadas, porém existem poucas evidências sobre seus fatores de risco potenciais.

**Objetivos:** Explorar os fatores de risco potenciais das lesões orais em cavalos trotadores Finlandeses.

**Delineamento experimental:** Estudo transversal.

**Métodos:** Examinou-se a porção rostral da boca de 261 cavalos (151 Standardbreds, 78 Finnhorses e 32 pôneis) após uma corrida de arreios. Foram coletadas informações sobre o tipo de embocadura, equipamento utilizado e desempenho na corrida.

**Resultados:** Um modelo de regressão logística multivariado utilizando dados obtidos em cavalos Standardbred e Finnhorse demonstrou maior risco de lesões orais moderadas ou graves em cavalos usando bridão maciço monoarticulado do tipo Crescendo (“Crescendo bit”) (n = 38, Razão de chances [OR] 3,6, Intervalo de Confiaça de 95% [CI] 1,4 - 8,9), bridão maciço não articulado do tipo Mullen (“Mullen mouth regulator bit”) (n = 25, OR 9,9, CI 2,2 - 45) ou bridão de plástico não articulado com meia colher (“straight plastic bit”) (n = 14, OR 13,7, CI 1,75 - 110) comparado com cavalos usando bridão maciço monoarticulado com meia colher (“snaffle trotting bit”) (n = 98, P = 0,002). As lesões na barra da mandíbula (67 cavalos) foram mais comuns em cavalos usando embocaduras não articuladas do que em cavalos usando embocaduras articuladas (teste exato de Fisher P < 0,001). As lesões na região vestibular e nas comissuras labiais internas não foram associadas ao tipo de embocadura. O uso de amarras de língua ou “overcheck”, galope, classificação entre os três primeiros ou premiação na corrida não foram associados com o risco de lesão.

**Principais limitações:** O tamanho amostral para certos tipos de embocaduras foi insuficiente para a análise estatística.

**Conclusões:** Lesões orais moderadas ou graves foram mais comuns em cavalos usando bridão monoarticulado maciço do tipo Crescendo, bridão não articulado maciço do tipo Mullen e bridão não articulado de plástico com meia colher do que em cavalos usando bridão monoarticulado maciço com meia colher. Todavia, as lesões foram observadas independentemente do tipo de embocadura. Estudos adicionais sobre a tensão da rédea, a interação entre o tipo de embocadura e a tensão da rédea e a prevenção de lesões orais em cavalos trotadores são necessários.
